# Epigenome-Wide DNA Methylation Levels During Pregnancy: Associations with Parity Across Diverse Populations

**DOI:** 10.3390/epigenomes10020030

**Published:** 2026-05-05

**Authors:** Su Chen, Yunsung Lee, Siri E. Håberg, Per Magnus, Christian Magnus Page, Emese H. C. Kovács, Anne L. Dunlop, Alicia K. Smith, John W. Holloway, Syed Hasan Arshad, Wilfried Karmaus, Susan L. Ewart

**Affiliations:** 1 Department of Biostatistics, University of Nebraska Medical Center, Omaha, NE 68198, USA; 2 Centre for Fertility and Health, Norwegian Institute of Public Health, Oslo 0213, Norway; yunsung.lee@fhi.no (Y.L.); sirieldevik.haberg@fhi.no (S.E.H.); per.magnus@fhi.no (P.M.); christianmagnus.page@fhi.no (C.M.P.); 3 Department of Global Public Health and Primary Care, University of Bergen, N-5020 Bergen, Norway; 4 Department of Physical Health and Ageing, Division of Public Health and Prevention, Norwegian Institute of Public Health, Oslo 0213, Norway; 5 Department of Gynecology and Obstetrics, School of Medicine, Emory University, Atlanta, GA 30322, USA; emese.kovacs@emory.edu (E.H.C.K.); amlang@emory.edu (A.L.D.); alicia.smith@emory.edu (A.K.S.); 6 School of Human Development & Health, Faculty of Medicine, University of Southampton, Southampton SO17 1BJ, UK; j.w.holloway@soton.ac.uk; 7 NIHR Southampton Biomedical Research Centre, University Hospital Southampton NHS Foundation Trust, Southampton SO16 6YD, UK; s.h.arshad@soton.ac.uk; 8 School of Clinical and Experimental Sciences, Faculty of Medicine, University of Southampton, Southampton SO17 1BJ, UK; 9 The David Hide Asthma and Allergy Research Centre, Isle of Wight, Newport PO30 5TG, UK; 10 Division of Epidemiology, Biostatistics, and Environmental Health, School of Public Health, University of Memphis, Memphis, TN 38152, USA; karmaus1@memphis.edu; 11 Department of Large Animal Clinical Sciences, College of Veterinary Medicine, Michigan State University, East Lansing, MI 48824, USA

**Keywords:** DNA methylation, epigenetics, pregnancy, parity, replication study, MoBa cohort, AAAMC cohort, IOW cohort

## Abstract

Background/Objectives: Parity, the number of times a woman carries a pregnancy to viability, has been linked to long-term maternal health outcomes. The mechanisms linking parity to health outcomes are poorly understood but may reflect influences of pregnancy on the maternal epigenome. Methods: This study examines the relationship between parity and DNA methylation (DNAm) during pregnancy using data from three cohorts: the Norwegian Mother, Father and Child Cohort Study (MoBa), the Atlanta African American Maternal-Child (AAAMC) cohort, and the Isle of Wight (IOW) Birth Cohort. Results: An epigenome-wide association study (EWAS) in MoBa identified 5374 cytosine–phosphate–guanine sites (CpGs) that were statistically significantly associated with parity, of which 69% were positively and 31% negatively correlated. Replication analyses confirmed 3491 CpGs in at least one cohort, and 93 CpGs in both AAAMC and IOW. Gene enrichment analysis revealed significant involvement of developmental and signaling pathways, including calcium signaling and neuroactive ligand–receptor interaction. Additionally, 584 differentially methylated regions (DMRs) were detected, with 90% overlapping individual parity-related CpGs. Conclusions: These findings suggest that parity influences epigenetic patterns, potentially affecting biological processes and molecular functions relevant to maternal health later in life.

## 1. Introduction

Parity, the number of times a woman carries a pregnancy to viability, has been shown to influence the likelihood of developing various health conditions in later life. A growing body of epidemiological evidence indicates that parity is associated with a wide range of disease risks and health outcomes, both beneficial and adverse. These include changes in the risk of cardiovascular disease [1,2,3,4], metabolic disorders such as type 2 diabetes [5,6,7,8,9], hormone-related cancers (including breast and ovarian cancers) [10,11,12], as well as autoimmune conditions [13,14,15], and mental health outcomes [16,17,18]. The relationship between parity and health is complex and likely shaped by a combination of biological, behavioral, and social mechanisms. Pregnancy induces profound physiological changes, including shifts in hormonal levels, immune modulation, and metabolic adaptations, many of which may have lasting effects beyond pregnancy and the postpartum period. Furthermore, cumulative exposures from multiple pregnancies may influence long-term disease risk through pathways such as inflammation, oxidative stress, endothelial function [19,20], and persistent fetal microchimerism [21].

Despite these associations, the biological mechanisms linking parity to health outcomes remain incompletely understood. Recent interest has turned to the role of epigenetics—particularly DNA methylation (DNAm)—as a potential mediator of these long-term effects. Epigenetic modifications offer a molecular framework through which pregnancy-related exposures could be biologically embedded, influencing gene regulation and disease susceptibility later in life. Our recent study identified 184 cytosine–phosphate–guanine sites (CpGs) that were significantly differentially methylated between parous and nulliparous women [22]. We further examined change in DNAm of these parous-related CpGs from pre-pregnancy to several years after giving birth in parous women compared to nulliparous controls over the same time interval and found that pregnancy and parturition may not only accelerate methylation changes but also slow down or halt methylation changes over time [23]. However, it is still unknown if the acceleration or deceleration of methylation changes in parous-related CpGs can be accumulated with multiple pregnancies. In this article, we conducted an epigenome-wide association study (EWAS) in blood-derived DNAm during pregnancy to identify CpG sites that are associated with parity.

## 2. Results

### 2.1. Population and Participant Characteristics

We analyzed the association between parity and DNAm in whole blood collected during pregnancy across three independent birth cohorts: the Norwegian Mother, Father and Child Cohort Study (MoBa), the Atlanta African American Maternal-Child (AAAMC) cohort, and the Isle of Wight (IOW) Birth Cohort. MoBa contains 114,151 observations during pregnancy, of which 2152 observations (2143 unique mothers) were analyzed in this EWAS. Some mothers had multiple pregnancies with at least one DNAm measurement. MoBa has the largest sample size and thus was considered the discovery cohort. A total of 676 observations from 416 unique mothers in the AAAMC cohort and 264 observations from 141 unique mothers in the IOW cohort were analyzed to replicate significant findings from the analyses of MoBa data. The distribution and summary statistics of demographics and characteristics of study populations and analyzed samples are provided in Table 1 and Table 2. Chi-square tests were performed to compare categorical characteristics between study populations and analyzed samples. Similar comparisons were performed for continuous characteristics with two-sample *t*-tests. Test statistics and *p*-values are reported in Table 1 and Table 2. The mean maternal ages were significantly different between study populations and analyzed samples in AAAMC and IOW. The distribution of parity and socioeconomic status (SES) were significantly different between the study population and the analyzed samples in MoBa. No other significant difference was observed in other characteristics in all three studies.

### 2.2. Epigenome-Wide Association Study (EWAS)

**Figure 1:**
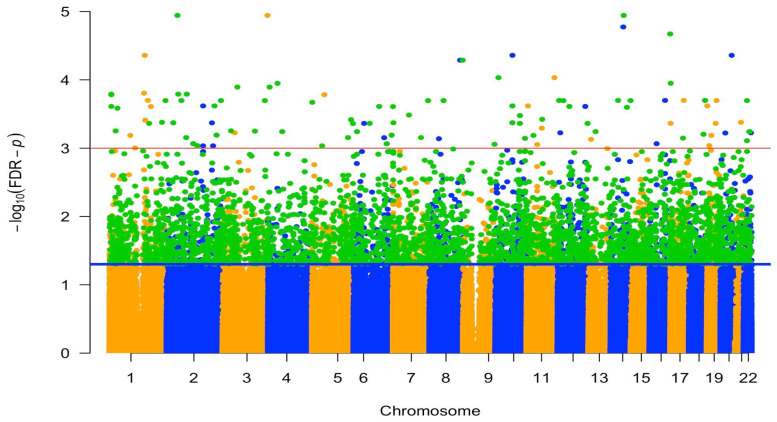
Differentially methylated positions identified in a Manhattan plot of the epigenome-wide association studies of parity in the MoBa cohort; orange and blue dots alternately represent CpG sites from chromosomes 1 through 22; dots above the blue line represent 5374 CpGs with FDR-*p* value less than 0.05; dots above the red line represent 110 CpGs with an FDR-*p* value less than 0.001. The green dots represent the 3491 CpGs that were replicated (and significantly associated with parity) in AAAMC (EPICv1 data) and/or IOW (intersect of EPICv1 and 450K data).

**Figure 2:**
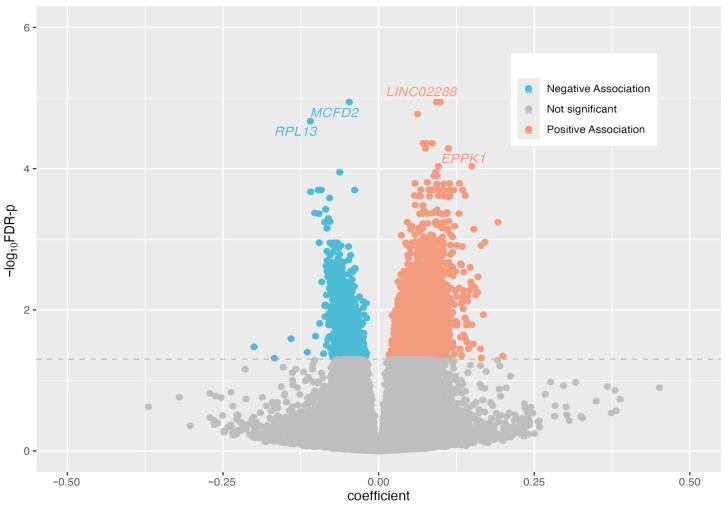
Volcano plot for all CpGs in the MoBa study. On the x-axis are the regression coefficients in the EWAS analysis of parity and y-axes is the negative log10-transformed FDR-adjusted *p*-values. Orange and blue dots represent the CpGs that were negatively and positively significantly associated with parity, respectively. The dashed line represents FDR-p=0.05. Among the top ten most significant CpGs identified in MoBa, four were replicated in IOW or AAAMC. The genes associated with these 4 CpGs are indicated in the plot. Gray dots represent the nonsignificant CpGs.

To investigate the epigenetic markers associated with parity, we conducted an EWAS using linear mixed models with repeated measures in MoBa, analyzing 769,139 CpG sites. CpGs with FDR adjusted *p*-value less than 0.05 were considered significantly associated with parity. We identified a total of 5374 parity-related CpGs associated with 2935 genes, represented by the dots above the blue line in the Manhattan plot (Figure 1). Among these 5374 CpGs, 3731 (69%) are positively associated with parity (orange dots in Figure 2) and 1643 (31%) are negatively associated with parity (blue dots in Figure 2).

### 2.3. Replication Analysis

**Figure 3:**
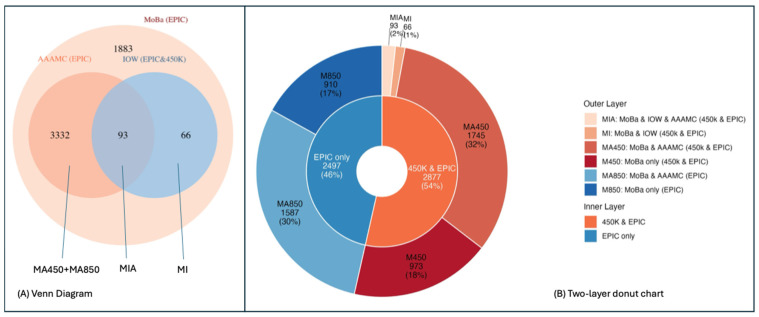
Venn diagram (**A**) and two-layer donut chart (**B**) demonstrating the overlapping parity-related CpGs among MoBa (discovery), AAAMC (replication), and IOW (replication) cohorts. MA450 + MA850 in (**A**) represents the total number of CpGs in MA450 and MA850 described in the legend of (**B**). MIA and MI in (**A**) are described in the legend of (**B**). “&” in (**A**) and (**B**) represents “and”.

Among the 5374 parity-related CpGs identified in MoBa, 3425 (64%) and 159 (3%) were replicated in AAAMC and IOW, respectively. Figure 3A presents a Venn diagram that depicts the number of overlapped parity-related CpGs among the three studies. The largest light orange circle represents the 5374 parity-related CpGs identified in MoBa. The 93 CpGs (2%) in the intersection were replicated in both AAAMC and IOW, while 3491 CpGs (65%) were replicated in either AAAMC or IOW. The regression coefficients, standard errors, and *p*-values for the top 10 replicated CpGs are reported in Table 3 (replicated twice) and Table 4 (replicated once), with the remainder of these data reported in the Supplemental Tables (Supplemental Tables.xlsx).

Methylation data for all samples in the MoBa and AAAMC cohorts were generated using Illumina MethylationEPICv1 arrays. For the IOW cohort, methylation profiling was performed using one of two platforms, with some samples assayed on Illumina 450K arrays and others on Illumina MethylationEPICv1 arrays; only CpGs that overlap between 450K and EPICv1 platforms were used in analyses of IOW data. Figure 3B stratifies the 5374 parity-related CpGs into two groups in the inner layer of the donut chart: (1) 2877 CpGs available in the IOW cohort, labeled “450K and EPIC”, representing CpGs present on both the 450K and EPICv1 platforms; and (2) 2497 CpGs not available in the IOW cohort, labeled “EPIC only”, representing CpGs included on the EPICv1 array but absent from the 450K array. The outer layer of the donut chart reports the number of CpGs replicated in the AAAMC and IOW cohorts within each group. Among 3332 CpGs only replicated in AAAMC (not IOW), 1587 (30% of 5374) CpGs were from the “EPIC only” group and 1745 (32% of 5374) CpGs were from the “450K and EPIC” group.

### 2.4. Functional Analysis

For gene enrichment analysis, the 3491 identified and replicated parity-related CpGs were annotated to 1993 unique genes and tested for enrichment of Kyoto Encyclopedia of Genes and Genomes (KEGG) pathways and Gene Ontogeny (GO) categories (biological process, cellular component, and molecular function) using GoMeth (missMethyl R package v1.40.3) [24]. Three KEGG pathways and 209 biological process, 25 cellular components, and 24 molecular function GO categories were identified (FDR *p*-value < 0.05). The three KEGG pathways are listed in Table 5. The top 10 results in each of the three GO categories are reported in Table 6.

For transcription factor binding analysis, we tested whether the top 10 replicated parity-associated CpGs reported in Table 3 (replicated twice) and Table 4 (replicated once) were enriched within transcription factor binding sites using eFORGE-TF [25]. Of the twenty CpGs analyzed, twelve (five of ten CpGs in Table 3 and seven of ten CpGs in Table 4) overlapped significantly with at least one transcription factor binding site (FDR-q value < 0.1). Detailed information on the overlapping transcription factor binding sites is provided in the Supplemental Tables (Supplemental Tables.xlsx).

### 2.5. Differentially Methylated Regions

In the MoBa study we identified 5374 individual parity-related CpGs. In addition, we used the DMRfinder (DMRff v1.1.2) R package [26] to identify 584 differentially methylated regions (DMRs), which are regional methylation alterations spanning clusters of CpG sites that collectively show consistent and statistically significant differential methylation. A total of 528 of the 584 (90%) identified DMRs contained at least one individual CpG site that was also found to be statistically significant in the EWAS analysis. Among the ten genes associated with the top ten parity-related CpGs reported in Table 4, six genes (*MCFD2*, *RPL13*, *SMG6*, *LIMCH1*, *ADAMTS9*, *NRXN1*) contain DMRs. To further validate whether the parity-associated CpGs exhibit measurable changes across the reproductive transition, we tested methylation changes of six significant CpGs (FDR-adjusted *p* < 0.05 in the EWAS) associated with three genes (*SMG6*, *ADAMTS9*, and *NRXN1*; significant CpGs associated with the other three genes were not available in the IOW data) from 28 parous women in IOW between pre-pregnancy at age 18 and post-parity at age 26 using two-sample paired *t*-tests. Four out of six (67%) analyzed CpGs were significantly differentially methylated between pre-pregnancy (age 18) and post-parity (age 28) with *p*-value < 0.05.

Table 7 displays the coefficient estimate, standard error, *p*-value, and associated gene of these six DMRs along with the number of individual parity-related CpGs contained in each DMR. The 584 significant DMRs were tested for KEGG pathways and GO categories (biological process, cellular component, and molecular function) using GoRegion (missMethyl v1.40.3 R package) [24]. One KEGG pathway and 45 biological processes, one cellular component, and 15 molecular function GO categories were identified (FDR *p*-value < 0.05). The full lists of 584 DMRs, and the KEGG pathways and GO categories associated with these DMRs, are reported in the Supplemental Tables (Supplemental Tables.xlsx).

## 3. Discussion

This study investigates the association between parity and DNAm during pregnancy using data from three cohorts: MoBa (Norway), AAAMC (Atlanta, US), and IOW (Isle of Wight, UK) and two different concepts: differently methylated positions (aka CpGs) and regions (DMRs). MoBa served as the discovery cohort with 2152 analyzed samples, while AAAMC and IOW were used for replication. In the primary EWAS analysis, 5374 CpGs were significantly associated with parity, with 69% positively and 31% negatively associated. Replication analysis confirmed 3491 parity-related CpGs in at least one cohort. Gene set enrichment analysis of these 3491 parity-related CpGs using GOmeth revealed three significant KEGG pathways and 258 GO categories related to development and signaling. Additionally, 584 DMRs were identified, most overlapping with individual parity-related CpGs. Among the genes annotated to the top 10 of 3491 identified and replicated CpGs, six genes (*MCFD2*, *RPL13*, *SMG6*, *LIMCH1*, *ADAMTS9*, and *NRXN1*) also contain at least one parity-associated DMR. Furthermore, eFORGE-TF analysis revealed enrichment of CpGs overlapping transcription factor binding sites, suggesting that these loci may reside in regulatory regions involved in transcriptional regulation. All these findings suggest that parity influences epigenetic patterns, potentially impacting biological processes and signaling pathways.

Our comprehensive approach has identified multiple genes and pathways, some with prior evidence of involvement in diseases for which parity modifies the risk, such as breast cancer (*LIMCH1*), type 2 diabetes (*ADAMTS9*), and neurodegenerative diseases (*NRXN1*). The *LIMCH1* gene is located within a parity-associated DMR and contains a parity-related CpG site in the MoBa study that was replicated in the AAAMC cohort. *LIMCH1* has been shown to be overexpressed in breast cancer [27]. It regulates nonmuscle myosin II (NM-II), which is a cytoskeletal protein involved in cell contractility and migration [28]. Through this mechanism, it may play a role in metastasis. In addition, *LIMCH1* increases mitogen-activated protein kinase (MAPK) activation with resultant upregulation of programmed death-ligand 1 (PD-L1). Binding of PD-L1 to programmed cell death protein 1 (PD-1) receptors on T-cells reduces T-cell activation and promotes immune evasion [29]. Evidence is mounting that these mechanisms are the way in which *LIMCH1* overexpression is permissive of breast cancer tumor growth and migration [30].

*ADAMTS9* is another gene located within a parity-associated DMR with an individual parity-associated CpG site that was identified in MoBa and replicated in AAAMC. This gene has been associated with type 2 diabetes in several studies [31,32,33] and it maps close (38 kb upstream) to the polymorphism rs4607103 that has been shown to decrease insulin sensitivity [34]; this mechanism is unique from pancreatic islet cell dysfunction. In a meta-analysis that excluded type 1 diabetes and gestational diabetes, parity was associated with type 2 diabetes [35].

*NRXN1* encodes neurexins, which are presynaptic proteins involved in synapse function and maintenance and play a key role in organizing active zones and regulating neurotransmitter release [36]. Its ligand, *NLGN1*, is required for glutamatergic synapse maturation and for the recruitment and stabilization of AMPA and NMDA receptors. Dysregulation of the neurexin–neuroligin complex alters receptor localization and synaptic efficacy, thereby modulating downstream ligand-receptor signaling cascades captured in the KEGG Neuroactive ligand–receptor interaction pathway [37,38]. Consistent with this functional role, deficits in *NLGN1* and *NRXN1* have been reported in neurodevelopmental and neuropsychiatric disorders [39] and in animal models of Alzheimer’s disease [36,40]. Although epidemiologic evidence linking parity to Alzheimer’s disease risk is mixed [16,41,42], our observation that parity-associated CpGs map to *NRXN1* and related synaptic organizers suggests that parity may induce durable epigenetic remodeling of synaptic ligand–receptor signaling networks.

Maternal age and parity are closely related and interact significantly, influencing both maternal and infant health outcomes [43,44]. A meta-analysis from the Pregnancy and Childhood Epigenetics (PACE) Consortium identified associations between maternal age and DNA methylation at 322 CpG sites in offspring [45]. Notably, only one of these 322 CpGs (cg07609862; Melatonin Receptor 1B, *MTNR1B*) overlapped with the 5374 parity-related CpGs identified in MoBa EWAS analyses, and this CpG was also replicated in the AAAMC cohort. In both discovery and replication analyses, models assessing parity associations with maternal DNAm were adjusted for maternal age, suggesting that parity may influence maternal DNAm independently of maternal age. The identification of *MTNR1B* as a CpG associated with both maternal age and parity highlights a potential epigenetic link between reproductive history and metabolic regulation. *MTNR1B* encodes the melatonin receptor 1B, which plays a central role in circadian regulation of insulin secretion and glucose homeostasis [46,47] and genetic variants in *MTNR1B* have been consistently associated with fasting glucose levels, gestational diabetes mellitus, and type 2 diabetes risk [48,49]. These findings suggest that both advancing maternal age and increasing parity may contribute to cumulative metabolic adaptations, including repeated periods of pregnancy-related insulin resistance and long-term alterations in glucose regulation, with *MTNR1B* potentially serving as a key molecular mediator of these processes. To further address whether parity-associated DNAm changes reflect general biological aging, we compared our 5374 parity-associated CpGs with 1062 CpGs included in established epigenetic clocks (353 from the Horvath clock [50], 71 from the Hannum clock [51], 513 from the PhenoAge clock [52], and 173 from the DunedinPACE [53]) estimated from blood samples. We identified only eight overlapping CpGs (cg06738602, cg08965235, cg06810647, ch.2.30415474F, cg21096399, cg07244253, cg00687674, cg24921089), indicating limited direct overlap. This suggests that, although previous studies have linked increasing parity with accelerated epigenetic aging, the CpGs we identified predominantly reflect parity-specific epigenetic effects rather than generalized age-related methylation changes.

This study has numerous strengths. First, the use of a large discovery cohort (MoBa) combined with replication in two independent cohorts (AAAMC and IOW) enhances the robustness and generalizability of findings. Second, the inclusion of diverse populations and repeated measures (early and/or late pregnancy for multiple pregnancies in the same women) further strengthens the validity of the observed associations. The discovery cohort (MoBa) and one of the replication cohorts (IOW) were of Northern European ancestry (White) while the other replication cohort was African American (AAAMC). Furthermore, all three cohorts were geographically distinct, originating from Western Europe, Northern Europe, or the United States of America. Thus, the replication of results across race and geography strengthens the generalizability of our findings. Additionally, the integration of epigenome-wide analyses with pathway enrichment provides biological context for the identified methylation changes.

Several limitations should also be noted. MoBa and IOW’s methylation data were generated from whole blood, while AAAMC were generated from PBMCs. In addition, although some circulating white blood cells have relatively short lifespans, others such as memory T- and B-cells are long lived. Furthermore, DNAm signatures measured in blood may reflect longer-term biological programming (e.g., at the level of hematopoietic stem or progenitor cells) rather than persistence within individual short-lived cells. Nevertheless, interpretation of blood-based methylation signals should be made cautiously. MoBa and AAAMC’s methylation data were generated exclusively on the EPICv1 platform. However, IOW only included the CpGs that overlapped between EPICv1 and 450K platforms to keep the maximum sample subjects. All analyzed subjects in the MoBa and IOW cohorts were live births, whereas 93% of analyzed subjects in the AAAMC cohort were live births. In a sensitivity analysis restricted to live births in AAAMC, 89% (3036 of 3425) CpGs remained successfully replicated. Furthermore, only linear relationships between parity and DNAm were investigated in this study. Prior studies have identified non-linear relationships (e.g., U-shape relationship) between parity and health outcomes later in life (e.g., Alzheimer’s Disease [54,55] and breast cancer [56]), which were not addressed in our analyses. This study focuses on the parity-associated individual CpGs and DMRs during pregnancy; external data are needed to validate whether these changes persist following pregnancy and have an impact on long-term health in women. Even though we adjusted for important covariates (e.g., gestational age, BMI, etc.) in our analyses, residual confounding by unmeasured factors such as diet, prenatal vitamins intake (e.g., folate), or environmental exposures cannot be ruled out. In addition, we did not assess whether identified methylation changes have downstream functional consequences on gene expression, which limits mechanistic interpretation. The information on DNAm did not include the X-chromosome, which may have been affected by parity and may affect maternal health status. We acknowledge that restricting analyses to autosomal CpGs may have excluded potentially informative X-chromosome signals. Since X-linked methylation is influenced by X-chromosome inactivation and variable escape from inactivation, dedicated analytical frameworks are needed for robust inference. Future investigations applying X-chromosome-specific modeling approaches may further elucidate sex-linked epigenetic mechanisms related to parity. Another limitation of this study is the lack of targeted validation of individual CpG sites using gene-specific assays such as pyrosequencing. However, replication of associations across three independent cohorts, with consistent effect directions and magnitudes, provides strong support for the findings. In addition, the Illumina Infinium methylation array has been extensively validated against quantitative bisulfite-based methods, demonstrating high concordance in CpG methylation measurements [57,58,59,60,61]. Together, these factors support the robustness of the reported CpG associations despite the absence of targeted experimental validation. Finally, we do not have complete information on whether intended or non-intended early loss of pregnancy may have an impact on DNAm of the participating women.

## 4. Materials and Methods

### 4.1. Study Populations

MoBa: The Norwegian Mother, Father and Child Cohort Study (MoBa) is a nationwide pregnancy cohort that enrolled approximately 95,000 mothers, 75,000 fathers, and 114,000 children across Norway between 1999 and 2008, corresponding to about 40% of invited pregnant women [62]. Fathers were included from 2001. Participants completed questionnaires during pregnancy and after childbirth and continue to be followed through repeated questionnaires and linkage with the Medical Birth Registry of Norway (MBRN). Blood samples were collected from mothers and fathers around gestational week 17, and at delivery from mothers (whole blood) and newborns (cord blood). We processed two sub-studies for DNAm measurements. (1) MoBa-START: we selected 992 mother–father–child trios with naturally conceived children and 978 trios with assisted reproductive technology (ART)-conceived children who fulfilled the following criteria: the children were singletons born between 2001 and 2009 with a birth record, the mothers had returned the first MoBa questionnaire at around 18 weeks of pregnancy, and DNA samples were available for all three family members. We only included the mothers who naturally conceived in our EWAS analyses. (2) MoBa-met008: we randomly selected 1200 mother–father–child trios with naturally conceived children. The selection criteria are the same as for MoBa-START. In total, 2152 analyzed samples (2143 unique mothers) from MoBa were used in the EWAS analysis.

AAAMC: The Atlanta African American Maternal-Child (AAAMC) cohort recruited pregnant women during their first trimester between 2014 and 2022 in Atlanta, Georgia [63]. Inclusion criteria were self-reported Black or African American women between 18 and 40 years old; able to give written informed consent; 8–14 weeks gestation with a singleton pregnancy (verified by electronic health record and/or ultrasound); no chronic health conditions (e.g., seizure disorders, chronic hypertension, diabetes mellitus); and no chronic prescription medication. Of the 416 participants, 1 participant had pregnancies that did not have birth outcomes (i.e., lost to follow up), 39 participants had an elective abortion or miscarriage, and 3 participants had a stillbirth. Blood samples were collected in EDTA-treated tubes at least one time (one at the first prenatal care visit occurring between 8 and 14 gestational weeks and the second at a later prenatal care visit occurring between 24 and 30 gestational weeks). Blood samples were processed to isolate peripheral blood monocular cells (PBMCs) prior to assay as described previously [64] and below.

IOW: The Isle of Wight (IOW) cohort is a multi-generational, population-based study in the United Kingdom to prospectively examine the development and progression of asthma, allergic disorders, and associated chronic diseases [65]. Study participants were enrolled in the birth cohort study between 1989 and 1990 by contacting potential parents (first generation, IOW-F0) while they were carrying their second-generation offspring (IOW-F1). The IOW-F1 participants have been followed up and assessed six times since birth up to the age of 26 years. Female participants and partners of the male participants of the IOW-F1 (*n* = 368) were recruited into the IOW 3rd generation study when they became pregnant and were monitored throughout their pregnancies, during which detailed information on their health and lifestyle was collected.

### 4.2. DNA Methylation Measurements and Cell Estimation

#### 4.2.1. DNA Methylation Measurements, Processing, and Quality Control

MoBa: DNA samples were shipped to the Institute of Life and Brain Sciences, University of Bonn, Germany (MoBa-START), and to Erasmus Medical Center, Rotterdam, the Netherlands (MoBa-met008) for processing and DNAm profiling using the Infinium MethylationEPICv1 BeadChip array (Illumina, San Diego, CA, USA) [60]. Bisulfite conversion was performed with the EZ-96 DNA Methylation-Lightning™ MagPrep kit (Zymo Research, Irvine, CA, USA), and raw data were generated using GenomeStudio 2011.2. iDAT files were processed separately by batch using the RnBeads package (v2.2.0) in R (v3.5.0) [66]. Quality control included removal of cross-hybridizing probes [67], probes with SNPs within the last three bases, and probes with high detection *p* values (>0.01). Batches were further filtered with the greedycut algorithm to exclude samples and probes showing outlying methylation profiles. Background correction was applied using ENmix.oob [68], and signal intensity was visually inspected via RnBeads control probe outputs. Any CpG site failing QC in one batch was excluded from all others, yielding 770,586 and 795,171 high-quality autosomal CpGs for analysis for MoBa-START and MoBa-met008, respectively. Finally, type I and type II probe distributions were normalized using Beta-mixture quantile normalization (BMIQ) [69] implemented in the watermelon package (v1.26.0) [70] in R (v3.5.0). A total of 769,139 common CpG sites were analyzed in the EWAS study.

AAAMC: Blood samples were collected in EDTA tubes at two timepoints, once between 8 and 14 weeks of pregnancy and again between 24 and 30 weeks. Samples were then centrifuged through a SepMate™ PBMC Isolation Tube (STEMCELL Technologies, Vancouver, BC, Canada). Isolated peripheral blood mononuclear cells (PBMCs) were then washed with PBS before storing them in PrepProtect™ Stabilization Buffer (Miltenyi Biotec, Bergisch Gladbach, Germany) at −80 °C. DNA was extracted from PBMCs with the AllPrep DNA/RNA Mini Kit (Qiagen, Germantown, MD, USA) and quantified with the Quanti-IT™ PicoGreen™ dsDNA assay (ThermoFisher, Waltham, MA, USA).

The DNAm data from these samples were measured using the Infinium HumanMethylationEPICv1 BeadChip (Illumina), then processed with a pipeline modified for EPICv1 analyses available on GitHub [71]. Briefly, the minfi v1.52.1 R package [72] was used to read in the iDATs and plotting the control plots for the RGset. The ewastools v1.7.1 package then computed detection *p*-values while screening for problematic samples (i.e., those that failed control metrics, were mislabeled, or duplicate samples) [73]. Enmix v1.46.1 [68,74] was used as a primary data processor: OOB (out-of-band) background correction and RELIC (REgression on Logarithm of Internal Control probes) dye-bias correction was implemented, then low quality samples and CpGs filtered out using a detection alpha of 0.05. After probe-type bias adjustment, polymorphic and off-target probe binding sites were omitted [67]. Another sanity check was conducted by predicting sex for each sample and the predicted sex was compared to the reported sex of each sample to ensure samples were not mislabeled. A total of 818,118 CpG sites were used for the replication analyses.

IOW: Peripheral whole blood samples were collected in IOW-F1 mothers during early (12 and 20 weeks) and/or late (28–32 weeks) pregnancy. DNA extraction was performed using either a standard salting procedure [75] or commercial kits (Qiagen) with DNA concentration determined by fluorometry (Qubit, Invitrogen™, Thermo Fisher Scientific). Then, DNA samples were assayed using either Infinium HumanMethylation450 arrays or Infinium MethylationEPICv1 BeadChips (Illumina) to measure epigenome-wide methylation levels. Multiple identical control samples were assigned to each bisulfite conversion batch for assessment of assay variability. CPACOR pipeline was used to preprocess DNA methylation data from both platforms (i.e., HumanMethylation450 and MethylationEPICv1), which includes background level correction and quantile normalization [76] implemented in a minfi R package [72]. Quantile-normalized intensities data were then used to calculate methylation levels in beta values defined as the proportions of intensity of methylated (M) over the sum of methylated and unmethylated (U) sites/probes (β = M/[c + M + U], where c is a constant to prevent zero in the denominator if M + U is too small). Since beta-values have severe heteroscedasticity for highly methylated or unmethylated CpG sites, base-2 logit-transformed beta values (denoted as M-values) have been recommended for the differential analysis of methylation levels [77]. For quality control purposes, probes that did not reach a *p*-value < 10^−16^ in at least 95% of samples were excluded. Similarly, samples with *p*-value > 10^−16^ in at least 95% of the CpGs were also excluded [76]. The HumanMethylation450 and MethylationEPICv1 arrays were initially pre-processed (as described above) separately for beta estimation and then beta values of common CpG sites (*n* = 389,355) between 450K and EPICv1 was merged for replication analyses. Batch effects were corrected using ComBat in R [78]. Since the third-generation cohort were recruited over years whenever female IOW-F1 members or partners of male IOW birth cohort members become pregnant, multiple pregnancies per IOW birth cohort women or men with different partners were collected and included in our analyses.

CpGs located on the X chromosome were excluded in this study. Due to X-chromosome inactivation (XCI) in females and resulting mosaic methylation patterns, X-linked CpGs exhibit distributional and variance characteristics that differ from autosomal loci and require specialized analytical approaches. As our preprocessing and modeling pipeline was optimized for autosomal CpGs, the primary analyses were restricted to autosomes to avoid potential bias.

#### 4.2.2. Cell Estimation

Previous studies have shown that DNAm profiles in whole blood are significantly affected by underlying cell type composition [79,80]. Therefore, adjusting for cell composition is essential in EWAS using peripheral whole blood or peripheral blood mononuclear cell DNAm data. Proportions of the cell types CD4^+^ T-cells (CD4T), CD8^+^ T-cells (CD8T), natural killer (NK) cells, B-cells, neutrophils, monocytes, and eosinophils were estimated using the Houseman’s approach [81] adapted by Jaffe and Irizarry [79] implemented through the “estimateCellCounts2” function from the R package minfi [72]. For EPIC arrays, the FlowSorted.Blood.EPIC reference panel [82] was used, while the FlowSorted.Blood.450k reference panel was applied for 450K arrays [83]. Estimated cell compositions were thus included as confounders in each cohort analysis.

### 4.3. Parity

Parity indicates the number of pregnancies reaching viability, commonly considered ≥20 weeks of gestation in U.S. obstetric practice and ≥24 weeks in some international settings, regardless of pregnancy outcome (live birth or stillbirth) [84,85]. In MoBa, parity was defined as the number of live births and stillbirths occurring at ≥12 weeks of gestation. However, only seven samples had stillbirths between 12 and 20 weeks included in their parity counting, indicating that the discrepancy between the U.S. and Norwegian gestational age thresholds unlikely influenced the analyses. All 2152 samples used for EWAS analyses from the MoBa cohort were live births. In total, 631 of 676 samples used for AAAMC replication analyses were live births. All 264 samples used for IOW replication analyses were live births. Terminated pregnancies or early lost pregnancies were not included in this study.

### 4.4. Confounding Variables

MoBa: Information on pre-pregnant BMI, pre-pregnant smoking status, age at menarche (in years), and maternal education was obtained from self-reported questionnaires. Implausible values for height and weight were set to missing before calculating BMI. Educational attainment was categorized into four groups: (1) less than high school, (2) high school, (3) some college, and (4) college or higher. Parity was retrieved from the Medical Birth Registry of Norway, while maternal age and gestational week at blood sampling were obtained from the internal database maintained by the MoBa Biobank.

AAAMC: Tobacco use within the past month was collected from medical records and by self-report with the Timeline Follow-back Interview (TLFB) [86]. Early pregnancy (from the first prenatal visit) BMI, gestational age, and parity were abstracted from the participants’ medical records. Sociodemographic characteristics (i.e., maternal age, maternal education level in four groups similar to MoBa groupings) were self-reported with a standardized interview questionnaire at the first study visit (8–14 weeks). More information on demographic and clinical questionnaires is described elsewhere [63,87,88].

IOW: Maternal age, pre-pregnant smoking status, and age at menarche (in years) were obtained from self-reported questionnaires. Pre-pregnant smoking was recorded as “yes” for a current smoker at age 18 before pregnancy. Pre-pregnant BMI was calculated from the height and weight at age 18. SES categories were generated by K-mean clustering using education, housing information, and income, which were extracted from the questionnaire. Education was recorded as “School”, “6th Form College”, “Further Education”, and “Other”. Housing information was recorded in ordinal data as “Rented Council/Housing Assoc.”, “Rented Private”, “Lives with Parents”, “Owned Private”, and “Other”. Income was also recorded in the ordinal groups as “Less than GBP 12,000”, “GBP 12,000 to 17,999”, “GBP 18,000 to 29,999”, “GBP 30,000 to 41,999”, “Greater than GBP 42,000”, and “Prefer not to say”. In the cluster analysis, “Other” in education and housing information, and “Prefer not to say” in the income were treated as missing values. PROC FASTCLUS was used to generate a cluster variable for the social economic status. Five clusters were chosen based on R2, Pseudo-F, and CCC statistics. The lowest SES cluster (=1) had low level of household income, and low number of rooms in the house, and low level of maternal education. The second SES cluster (=2) had low level of household income, low number of rooms, and low-to-medium level of maternal education. The third SES cluster (=3) had low-to-medium level of household income, low number of rooms, and high level of maternal education. The fourth SES cluster (=4) had medium level of household income, high number of rooms, and low-to-medium level of maternal education. The highest SES cluster (=5) had high level of household income, high number of rooms in the house, and medium level of maternal education.

In all three cohorts (MoBa, AAAMC, and IOW), confounding variables included mother’s age at the pregnancy, pre-pregnant BMI, (active) smoking status, and maternal SES or education levels. Gestational age in weeks was adjusted as a confounding variable only in AAAMC and IOW data analyses since all MoBa DNAm samples were collected in 17–18 weeks of pregnancy. Age at menarche was adjusted as a confounding variable only in MoBa and IOW data analyses (AAAMC did not collect “age at menarche”).

### 4.5. Statistical Analysis

We conducted an EWAS of parity to identify CpGs that are significantly differentially methylated during pregnancy in women who naturally conceived in the MoBa cohort. To account for potential batch effects, we converted the quality-controlled DNAm beta-values into M-values and applied the ComBat function [78]. The linear mixed model analyses were implemented using the lme function from the nlme R package [89] for the discovery analyses with MoBa data and using the lme4 v1.1.38 [90] and lmerTest v3.2.0 R packages [91] for the replication analyses with AAAMC and IOW data. Random intercepts were included in the model to account for the chip batch effect. Cell compositions (CD8T, CD4T, NK, B-cell, monocytes, and neutrophils), maternal age (i.e., age at pregnancy), active and passive (i.e., second-hand) smoking (3 months before pregnancy), BMI before pregnancy, education, and age at menarche were adjusted as covariates. All methylation samples in MoBa were collected at 17–18 gestational weeks and thus gestational age did not need to be adjusted for the analysis. Correction for multiple testing was carried out using the Benjamini Hochberg procedure (FDR method) [92]. In addition to the identification of individual parity-associated CpGs, we performed regional DNAm analyses using DMRff [26] to identify DMRs associated with childbirth in women. EWAS and DMRff capture complementary aspects of DNA methylation variation. While EWAS evaluates associations at individual CpG sites, DMRff leverages spatial correlation among neighboring CpGs to identify differentially methylated regions that may reflect more coordinated and biologically meaningful epigenetic changes; therefore, applying both approaches provides a more comprehensive characterization of exposure-associated methylation patterns. Following these analyses, gene set enrichment analyses were performed using GOmeth and GOregion methods (for differentially methylated individual CpGs and DMRs) implemented in the “missMethyl” (v1.40.3) Bioconductor R package [24]. *p*-values for enrichment were adjusted for multiple testing using the FDR method [92]. To explore the potential functional relevance of parity-associated CpG sites, we performed transcription factor binding enrichment analysis using eFORGE-TF, implemented within eFORGE v2.0 [25]. This analysis evaluates whether EWAS CpG sites are enriched within transcription factor binding regions derived from large-scale epigenomic datasets, including ChIP-seq data from the ENCODE Project. The method compares the observed overlap of CpG sites with transcription factor binding sites against matched background probe sets while accounting for genomic features of the array probes. Statistical significance was assessed using background permutations with false discovery rate (FDR) correction for multiple testing.

We replicated the identified CpGs in two independent cohort studies: AAAMC and IOW. The same linear mixed model was implemented with DNAm during pregnancy as the dependent variable and parity as the predictor. The same sets of covariates were adjusted in the model, except for (i) gestational age in weeks was also adjusted in IOW and AAAMC and (ii) age at menarche was only adjusted in MoBa and IOW. For the smaller replication cohort (*n* = 264), we conducted a power analysis based on a linear regression model including 11 predictors. Using a moderate effect size of f^2^ = 0.12, a significance level of 0.05 and a conservative Bonferroni adjustment for the 5374 CpGs identified in the discovery cohort, the replication cohort had 84% power to detect the reported associations. This analysis demonstrates that the replication cohort was adequately powered to validate the top findings from the discovery cohort.

## 5. Conclusions

In summary, we identified and replicated thousands of parity-associated CpG sites during pregnancy across ancestrally and geographically diverse populations. These findings suggest that parity is associated with widespread epigenomic variation in the maternal methylome and implicate pathways that may be relevant to long-term maternal health. Future studies will be needed to examine whether these methylation signatures persist after pregnancy, influence gene expression, and/or mediate disease risk. Future studies are also indicated to additionally assess DNAm on the X-chromosome and to investigate whether early pregnancy loss may also have affected maternal DNAm. Longitudinal and mechanistic work integrating multi-omics and clinical outcomes will be essential to determine the functional and clinical relevance of these epigenetic patterns.

## Figures and Tables

**Table 1:** Descriptive characteristics of study populations and analyzed samples.

Characteristics		MoBa			AAAMC			IOW		
		Study Population	Analyzed Samples	Test Stat (*p*-Value)	Study Population	Analyzed Samples	Test Stat (*p*-Value)	Study Population	Analyzed Samples	Test Stat (*p*-Value)
Sample size *		114,151 (94,742 unique mothers)	2152 (2143 unique mothers)	-	1128 (702 unique mothers)	676 (416 unique mothers)	-	617 (368 unique mothers)	264 (141 unique mothers)	-
Parity ^++^ (number of live births; see Section 4.3 for more details)	1	50,017	1009	8.8 (0.01) **	513	298	0.6 (0.74) **	318	125	3.2 (0.2) **
	2	40,834	755		310	197		195	92	
	3+	22,824	388		305	181		82	46	
	NA	476	0		0	0		22	1	
Maternal age (yrs)	Mean (SD)	30.1 (4.6)	30.1 (4.4)	0 (1)	26.1 (5.0)	25.5 (4.8)	2.5 (0.01)	25.3 (4.0)	24.3 (1.9)	5.0 (<0.001)
Smoking status before pregnancy	Yes	29,011	611	0.35 (0.55) **	205	131	0.33 (0.57) **	153	99	0.29 (0.59) **
	No	61,816	1342		923	545		260	153	
	NA	23,324	199		0	0		204	12	
BMI before pregnancy	Mean (SD)	24.1 (4.3)	24.2 (4.4)	−1 (0.30)	29.1 (8.07)	28.7 (7.96)	1 (0.30)	24.7 (4.6)	25.1 (4.70)	−1.2 (0.24)
Gestational age ^+^ (weeks)	Mean (SD)	18.0 (1.5)	18.0 (1.5)	0 (1)	17.1 (7.8)	17.4 (7.8)	−0.79 (0.43)	21.5 (8.4)	21.2 (8.6)	0.48 (0.63)
Age at Menarche (yrs)	Mean (SD)	13.0 (1.4)	13.0 (1.3)	0 (1)	NA	NA	NA	12.6 (1.5)	12.6 (1.5)	0 (1)

* One measurement during each pregnancy was counted as one observation sample. ** Missingness was not included in the chi-square test. ^+^ Gestational age when the DNAm measurement was taken. ^++^ Parity included the current pregnancy.

**Table 2:** Maternal socioeconomic status (SES) of study populations and analyzed samples.

	Maternal Education/SES*	Study Population	Analyzed Samples	Test Stat (*p*-Value)
MoBa	1: Less than high school	8257	133	12.1 (0.007) **
	2: High school	28,734	582	
	3: Some college ^+^	41,435	889	
	4: College and higher	22,981	512	
	NA	12,744	36	
AAAMC	1: Less than high school	161	99	2.4 (0.5)
	2: High school	438	273	
	3: Some college	322	199	
	4: College graduate or above	207	105	
IOW	1—Lowest SES*	76	48	3.2 (0.52) **
	2	97	73	
	3	113	72	
	4	60	46	
	5—Highest SES*	49	23	
	NA	222	2	

^+^ The category “Some college” includes degrees shorter than four years, such as medical technician. * SES categories were generated from education levels, housing information and income. More details on how SES categories were generated can be found in Section 4.4. ** Missingness was not included in the chi-square test.

**Table 3:** Top 10 parity-related CpGs that were statistically associated with parity in the MoBa study and both replication studies (AAAMC and IOW).

CpGs	MoBa			AAAMC		IOW		CHR	MAPINFO	Gene Name	Relation to CpG Island *	Same Coefficient Direction ^+^
	b (SE)	*p*	FDR.*p*	b (SE)	*p*	b (SE)	*p*					
cg16272981	−0.11 (0.02)	1.1 × 10^−8^	2.1 × 10^−4^	−0.05 (0.03)	4.0 × 10^−2^	−0.08 (0.03)	3.0 × 10^−2^	5	1489889	*LPCAT1*	Island	Y
cg26405097	0.05 (0.01)	5.9 × 10^−8^	5.7 × 10^−4^	0.06 (0.02)	5.6 × 10^−3^	0.04 (0.01)	4.8 × 10^−3^	6	15428301	*JARID2*		Y
cg25984344	0.15 (0.03)	9.0 × 10^−8^	7.2 × 10^−4^	0.18 (0.05)	2.6 × 10^−4^	0.11 (0.05)	4.2 × 10^−2^	10	128994608	*DOCK1*; *FAM196A*	Island	Y
cg14603031	0.07 (0.01)	1.8 × 10^−7^	1.1 × 10^−3^	0.05 (0.02)	3.7 × 10^−3^	0.05 (0.02)	6.5 × 10^−3^	7	2563184	*LFNG*	N_Shore	Y
cg03181582	0.06 (0.01)	2.0 × 10^−7^	1.2 × 10^−3^	0.05 (0.02)	3.6 × 10^−2^	0.05 (0.02)	1.4 × 10^−2^	11	17498530	*ABCC8*	Island	Y
cg20301308	0.10 (0.02)	2.2 × 10^−7^	1.2 × 10^−3^	0.07 (0.03)	2.7 × 10^−2^	0.05 (0.02)	2.3 × 10^−2^	1	65534742	*JAK1*	S_Shore	Y
cg05757530	−0.07 (0.01)	2.2 × 10^−7^	1.2 × 10^−3^	−0.07 (0.02)	2.8 × 10^−3^	−0.04 (0.01)	2.9 × 10^−3^	16	57038916	*NLRC5*		Y
cg03129384	0.10 (0.02)	3.4 × 10^−7^	1.5 × 10^−3^	0.10 (0.03)	7.9 × 10^−4^	0.05 (0.02)	3.1 × 10^−2^	10	128994644	*FAM196A*; *DOCK1*	Island	Y
cg14049470	0.09 (0.02)	5.4 × 10^−7^	2.0 × 10^−3^	0.11 (0.03)	8.1 × 10^−5^	−0.05 (0.02)	3.8 × 10^−2^	10	20105452	*PLXDC2*	Island	N
cg08891071	0.05 (0.01)	1.2 × 10^−6^	2.9 × 10^−3^	0.04 (0.01)	5.4 × 10^−3^	0.03 (0.02)	4.3 × 10^−2^	5	150399909	*GPX3*	N_Shore	Y

b: regression coefficients; SE: standard error; *p*: *p*-value; FDR.*p*: FDR (false discovery rate) adjusted *p*-value; CHR: chromosome; MAPINFO: genomic position (GRCh37-based). * Per UCSC Genome Browser CpG islands track settings. ^+^ Coefficients from CpGs that were not statistically significant (*p*-value > 0.05) or not available in a cohort were not checked for direction. Annotation was obtained from the Infinium MethylationEPIC v1.0 B5 Manifest File.

**Table 4:** Top 10 parity-related CpGs that were statistically associated with parity in MoBa study and at least one of the two replication studies (AAAMC or IOW).

CpGs	MoBa			AAAMC		IOW		CHR	MAPINFO	Gene Name	Relation to CpG Island *	Same Coefficient Direction ^+^
	b (SE)	*p*	FDR.*p*	b (SE)	*p*	b (SE)	*p*					
cg10499126	−0.05 (0.01)	1.6 × 10^−11^	1.1 × 10^−5^	−0.04 (0.01)	8.9 × 10^−4^	NA	NA	2	47137142	*MCFD2*		Y
cg24774357	0.09 (0.01)	4.4 × 10^−11^	1.1 × 10^−5^	0.09 (0.02)	2.7 × 10^−4^	0.02 (0.02)	0.19	14	77504364	*LINC02288*		Y
cg25145728	−0.11 (0.02)	1.4 × 10^−10^	2.1 × 10^−5^	−0.08 (0.02)	6.3 × 10^−4^	NA	NA	16	89628775	*RPL13*	S_Shore	Y
cg08628757	0.11 (0.02)	6.7 × 10^−10^	5.2 × 10^−5^	0.08 (0.03)	2.2 × 10^−3^	NA	NA	8	144938596	*EPPK1*	N_Shore	Y
cg20207763	0.15 (0.02)	1.4 × 10^−9^	9.3 × 10^−5^	0.12 (0.04)	3.8 × 10^−3^	0.02 (0.03)	0.48	10	13392088	*SEPHS1*		Y
cg19293468	−0.06 (0.01)	1.9 × 10^−9^	1.1 × 10^−4^	−0.13 (0.03)	1.4 × 10^−5^	−0.02 (0.02)	0.37	17	1973400	*SMG6*		Y
cg26248284	0.09 (0.02)	2.0 × 10^−9^	1.1 × 10^−4^	0.10 (0.03)	2.0 × 10^−4^	−0.01 (0.02)	0.76	4	41362853	*LIMCH1*	Island	Y
cg08273640	0.09 (0.01)	2.7 × 10^−9^	1.3 × 10^−4^	0.06 (0.02)	2.3 × 10^−2^	0.02 (0.02)	0.18	4	6909718	*TBC1D14*	N_Shore	Y
cg21878275	0.09 (0.02)	2.7 × 10^−9^	1.3 × 10^−4^	0.13 (0.04)	2.5 × 10^−3^	NA	NA	3	64673501	*ADAMTS9*	Island	Y
cg14875171	0.13 (0.02)	3.8 × 10^−9^	1.6 × 10^−4^	0.19 (0.05)	3.8 × 10^−5^	0.01 (0.03)	0.73	2	50574196	*NRXN1*	Island	Y

b: regression coefficients; SE: standard error; *p*: *p*-value; FDR.*p*: FDR (false discovery rate) adjusted *p*-value; CHR: chromosome; MAPINFO: genomic position (GRCh37-based). * Per UCSC Genome Browser CpG islands track settings. ^+^ Coefficients from CpGs that were not statistically significant (*p*-value > 0.05) or not available in a cohort were not checked for direction. Annotation was obtained from the Infinium MethylationEPIC v1.0 B5 Manifest File.

**Table 5:** KEGG pathways associated with 3491 replicated parity-related CpGs.

Pathway Name	Total Number of Genes in the Pathway	Number of Identified Genes in the Pathway	*p*-Value	FDR Adjusted *p*-Value
Neuroactive ligand–receptor interaction	366	54	1.8 × 10^−5^	6.7 × 10^−3^
Calcium signaling pathway	252	54	1.4 × 10^−4^	2.6 × 10^−2^
Neuroactive ligand signaling	199	41	2.8 × 10^−4^	3.5 × 10^−2^

**Table 6:** Top 10 biological process (BP), cellular component (CC), and molecular function (MC) Gene Ontology categories associated with 3491 replicated parity-related CpGs.

Category	Gene Function	*N*	DE	P.DE	FDR
BP	multicellular organism development	4818	725	5.1 × 10^−20^	1.1 × 10^−15^
BP	system development	4151	649	1.2 × 10^−19^	1.3 × 10^−15^
BP	anatomical structure morphogenesis	2780	478	6.3 × 10^−19^	4.7 × 10^−15^
BP	anatomical structure development	6065	839	1.1 × 10^−17^	5.9 × 10^−14^
BP	developmental process	6626	879	9.9 × 10^−16^	4.4 × 10^−12^
BP	nervous system development	2618	443	2.5 × 10^−14^	9.5 × 10^−11^
BP	multicellular organismal process	7447	916	4.7 × 10^−14^	1.5 × 10^−10^
BP	animal organ morphogenesis	1019	205	7.6 × 10^−14^	2.1 × 10^−10^
BP	animal organ development	3073	458	5.0 × 10^−12^	1.2 × 10^−8^
BP	tube development	1187	211	1.3 × 10^−11^	3.0 × 10^−8^
CC	cell periphery	6016	751	2.3 × 10^−10^	3.2 × 10^−7^
CC	synaptic membrane	452	108	1.1 × 10^−9^	1.1 × 10^−6^
CC	cell junction	2381	390	6.3 × 10^−9^	3.6 × 10^−6^
CC	glutamatergic synapse	584	125	6.4 × 10^−8^	2.4 × 10^−5^
CC	somatodendritic compartment	859	163	7.6 × 10^−8^	2.7 × 10^−5^
CC	synapse	1656	281	9.9 × 10^−8^	3.4 × 10^−5^
CC	plasma membrane	5536	673	1.5 × 10^−7^	5.1 × 10^−5^
CC	dendrite	629	128	2.6 × 10^−7^	7.9 × 10^−5^
CC	dendritic tree	631	128	2.8 × 10^−7^	8.3 × 10^−5^
CC	postsynaptic membrane	320	74	1.9 × 10^−6^	5.0 × 10^−4^
MF	cis-regulatory region sequence-specific DNA binding	1191	183	8.4 × 10^−10^	8.9 × 10^−7^
MF	RNA polymerase II cis-regulatory region sequence-specific DNA binding	1168	178	1.3 × 10^−9^	1.1 × 10^−6^
MF	DNA-binding transcription factor activity	1427	204	1.3 × 10^−9^	1.1 × 10^−6^
MF	DNA-binding transcription factor activity, RNA polymerase II-specific	1340	194	2.1 × 10^−9^	1.6 × 10^−6^
MF	sequence-specific double-stranded DNA binding	1535	220	2.5 × 10^−9^	1.7 × 10^−6^
MF	RNA polymerase II transcription regulatory region sequence-specific DNA binding	1371	196	6.8 × 10^−9^	3.7 × 10^−6^
MF	transcription cis-regulatory region binding	1470	210	7.7 × 10^−9^	4.1 × 10^−6^
MF	transcription regulatory region nucleic acid binding	1471	210	8.4 × 10^−9^	4.3 × 10^−6^
MF	signaling receptor activity	1473	176	1.5 × 10^−8^	6.8 × 10^−6^
MF	molecular transducer activity	1473	176	1.5 × 10^−8^	6.8 × 10^−6^

*N* = number of genes in the GO term, DE = number of genes differentially methylated, P.DE = *p*-value for over-representation of the GO term, FDR = false discovery rate.

**Table 7:** Parity-associated DMRs that are in the same genes among the top 10 parity-related CpGs in the MoBa study.

Chr	Start	End	#number of CpGs	Estimate	se	*p*-Value	FDR*p*	Gene
2	47137142	47137221	2	−3.32	0.43	1.95 × 10^−14^	1.82 × 10^−8^	*MCFD2*
16	89628775	89628775	1	−1.13	0.18	2.95 × 10^−10^	2.75 × 10^−4^	*RPL13*
17	1973400	1973400	1	−0.59	0.10	3.79 × 10^−9^	3.54 × 10^−3^	*SMG6*
4	41362853	41362853	1	2.67	0.45	4.09 × 10^−9^	3.81 × 10^−3^	*LIMCH1*
3	64670640	64672197	9	0.89	0.15	5.43 × 10^−9^	5.06 × 10^−3^	*ADAMTS9*
2	50574196	50574196	1	20.65	3.57	7.45 × 10^−9^	6.95 × 10^−3^	*NRXN1*

Assembly UCSC Genome Browser on Human (GRCh37/hg19). Chr: chromosome; estimate: coefficient estimate; FDR*p*: FDR adjusted *p*-value.

## Data Availability

Access to the MoBa genetic datasets can be obtained by applying to the Norwegian Institute of Public Health (NIPH; http://www.fhi.no/en/,https://www.fhi.no/en/studies/moba/ (accessed on 1 November 2025)). Restrictions may apply regarding the availability of these data, which were originally used under specific approvals for the current study and are therefore not publicly available. Access can only be given after approval by REK under the provision that the applications are consistent with the consent provided. An application form can be found on the NIPH website at https://www.fhi.no/en/studies/moba/ (accessed on 1 November 2025). Specific questions regarding access to data in MoBa study can also be directed to Dr. Siri E. Håberg (sirieldevik.haberg@fhi.no). Access to the AAAMC cohort data can be obtained with an approved data analysis plan submitted to Drs. Dunlop (amlang@emory.edu) or Smith (alicia.smith@emory.edu). Information on IOW data access is available from this link: https://allergyresearch.org.uk/studies/birth-cohort/#cohort-data-use (accessed on 4 December 2025).

## Supplementary Materials

The following supporting information can be downloaded at: https://www.mdpi.com/article/10.3390/epigenomes10020030/s1. Table S1: Parity-related CpGs that were statistically associated with parity in the MoBa study and both replication studies (AAAMC and IOW). Table S2: Parity-related CpGs that were statistically associated with parity in MoBa study and at least one of the two replication studies (AAAMC or IOW). Table S3: 584 DMRs that were statistically associated with parity in MoBa study. Table S4: Biological process (BP), cellular component (CC), and molecular function (MC) Gene Ontology categories associated with 3491 replicated parity-related CpGs. Table S5: KEGG pathways associated with 584 significant DMRs. Table S6: Biological process (BP), cellular component (CC), and molecular function (MC) Gene Ontology categories associated with 584 significant DMRs. Table S7: Transcription factor binding sites that are significantly overlapped with the top 10 replicated pari-ty-associated CpGs reported in Table 3 and Table 4.
